# A multi-objective optimization evaluation model for seismic performance of slopes reinforced by pile-anchor system

**DOI:** 10.1038/s41598-024-55766-3

**Published:** 2024-02-29

**Authors:** Lei Xue, Longfei Li, Chao Xu, Yuan Cui, Hao Ding, Kun Huang, Zhuan Li

**Affiliations:** 1 Key Laboratory of Shale Gas and Geoengineering, Institute of Geology and Geophysics, Chinese Academy of Sciences, Beijing, 100029 China; 2 Innovation Academy for Earth Science, Chinese Academy of Sciences, Beijing, 100029 China; 3 College of Earth and Planetary Sciences, University of Chinese Academy of Sciences, Beijing, 100049 China; 4 College of Geosciences and Engineering, North China University of Water Resources and Electric Power, Zhengzhou, 450046 China; 5 School of Engineering and Technology, China University of Geosciences (Beijing), Beijing, 100083 China

**Keywords:** Slope, Pile-anchor system, Reinforcement effect, Seismic performance, Multi-objective optimization evaluation model, Natural hazards, Solid Earth sciences

## Abstract

The significance means of the seismic reinforcement effect of a pile-anchor system for slope reinforcement has been widely recognized. However, cases of deformation failure and instability sliding of the pile-anchor system itself and the reinforced slope under seismic action continue to be recorded. Therefore, it is crucial to evaluate the seismic performance of slopes reinforced by a pile-anchor system to prevent the system’s failure. Current evaluation models of a slope reinforced by a pile-anchor system mainly focus on slope stability; however, the safety of the pile-anchor system itself is not sufficiently considered in these models. Consequently, in this study, we propose a multi-objective optimization evaluation (*MOE*) model for evaluating the seismic performance of slopes reinforced by a pile-anchor system that considers slope stability, safety of the pile-anchor system, and dynamic response of the slope. This model considers slope displacement, acceleration amplification factor of a slope, pile displacement, and anchor displacement as negative indexes, and anti-slide pile bending moment, shear force, and anchor axial force as intermediate indexes. The comprehensive weight of relevant indexes is obtained by combining subjective and objective weights, and the seismic reinforcement effect of the pile-anchor system is evaluated subsequently. In conclusion, the *MOE* model proposed in this study provides a novel solution for the optimization evaluation of a slope reinforced by a pile-anchor system in forthcoming projects.

## Introduction

Southwest China has a complex geological structure, variable topography and geomorphology, a large number of slopes are distributed in the alpine and valley areas, that are highly susceptible to deformation and instability during earthquake activity^1–3^. In recent years, several earthquakes have occurred in southwestern China (some examples include the Wenchuan earthquake that struck on May 12, 2008 in Sichuan; the Ludian earthquake, which struck on August 3, 2014 in Yunnan; and the Luding earthquake that struck on September 5, 2022 in Sichuan, etc.). These earthquakes have triggered numerous seismic landslides^4–6^, and have caused significant harm to the lives and properties of the local people. Therefore, introducing seismic reinforcement design to counter landslides holds considerable importance, and numerous methods for seismic reinforcement have been successively proposed^7–9^. Among these, the effectiveness of the seismic performance of the pile-anchor system has been widely recognized after several earthquakes, and has become one of the main methods of slope reinforcement in strong earthquake-prone areas. However, many cases of deformation failure and instability sliding of the pile-anchor system itself and the reinforced slope during earthquake activity continue to be recorded. For example, during the Wenchuan earthquake, 25 cases of instability of reinforced slopes were recorded in the Yingxiu-Wenchuan section of National Road213 alone^10^. In such instances, it is crucial to simultaneously consider the slope’s stability and the pile-anchor system’s safety when performing seismic reinforcement design optimization evaluation. It is important to note that various factors can affect the stability of the reinforced slope and the safety of the pile-anchor system^11^, including interactions between the slope and pile-anchor system as well as pile-anchor system parameters (for example, pile length, pile location, anchor length, anchor angle, anchor row spacing, etc.).

Previous studies have predominantly adopted Newmark analysis or the limit equilibrium method, taking the permanent slope displacement or stability coefficient as the only index to evaluate the stability of reinforced slopes^12,13^. Although the aforementioned studies have provided the corresponding design parameters, they have ignored considering the safety of the pile-anchor system itself and failed to fully consider the complexity of seismic loading. Large-scale shaking table tests constitute an important approach to study the dynamic response of slopes; these tests have been employed by some scholars to analyze the seismic performance of slopes under pile-anchor support, resulting in many useful conclusions^14–16^. However, due to the differences in the properties of similar materials, the existence of size effects, and the inconsistency between boundary conditions and reality, the results of shaking table tests often deviate from reality. Moreover, the high cost of conducting these tests also makes it difficult to perform extensive shaking table tests. Both the theoretical analysis and experimental methods as mentioned above belong to noncoupled analysis methods, and the earth pressure and its distribution obtained through these methods can be easily used to analyze the mechanical properties of a pile-anchor system and the stability of a reinforced slope; however, the interaction between pile-anchor structures and slope is not considered sufficiently in previous studies. In contrast, the numerical simulation method enables direct analysis of the deformation, stress, and stability of both the pile-anchor structure and the reinforced slope under seismic action through coupled analysis^17,18^; as it fully considers the interaction between the reinforced structure and slope, it is a more ideal evaluation method.

Yao et al.^19^ proposed a multi-objective optimization design framework for landslide reinforcement with anti-slide piles by considering the Majiagou landslide reinforced with anti-slide piles as a case study. They evaluated the stability of the landslide-pile system, and employed the Pareto optimal method to obtain the optimal design scheme. Based on limit analysis and the pseudo-dynamic method, Yan et al.^20^ proposed a slope stability evaluation method that considers the dynamic change in the anchor cable axial force under earthquake conditions. Li and Xiao^21^ used the upper bound theorem combined with the pseudo-static method to analyze the seismic stability of a slope reinforced with single-row piles by considering the axial force of the pile, subsequently analyzing the influence of the axial force of the pile on the potential sliding mass. Nazari and Ghanbari^22^ designed a variety of configurations of anti-slide piles, and employed the response surface method considering soil spatial variability and seismic action to evaluate the stability of the slope reinforced by single-row piles. The abovementioned studies have achieved certain results regarding the evaluation of seismic reinforcement of slopes; however, most of them are aimed at only one type of structure; however, in reality, combined reinforcement of piles and anchors is employed; thus, these studies fail to consider the synergistic reinforcement of pile-anchor structures.

Regarding the evaluation of a pile-anchor reinforced slope, Chen et al.^23^ decomposed the failure events of a pile-anchor structure reinforced slope into multiple anchor pile failure events with different numbers and sequences, and proposed a new reliability evaluation method for the pile-anchor reinforced slope system. Huang et al.^24^ performed a simulation of a pile-anchor reinforced slope by considering the spatial variability of soil properties by using they Monte Carlo method, and proposed a multiobjective optimization design framework for a slope reinforced by a pile-anchor structure based on the Pareto optimal theory. Xu and Huang^25^ studied the seismic stability of a slope reinforced by a pile-anchor structure under different structural parameters, and evaluated the seismic stability of the slope by combining dynamic finite element analysis with the Newmark permanent displacement method. Li and Wang^26^ simulated the slope under pile-anchor support by using the three-dimensional finite element method; they analyzed the effect of seismic load on the deformation of the pile-anchor structure, and proposed the concept of a pile-anchor supporting coordinate interval to evaluate the stability of the slope. Huang et al.^27^ compared and analyzed the dynamic response of a slope under four reinforcement schemes by using the finite element method, and found that the pile-anchor structure has a significant seismic advantage as compared to other structures; based on the Newmark permanent displacement method, they proposed a new calculation method for the slope safety factor to evaluate the seismic stability of the slope. In summary, most existing studies only focus on slope deformation and stability, ignoring the evaluation of the safety of the pile-anchor system itself. Additionally, the dynamic response of the pile-anchor structure is insufficiently considered in these studies.

In general, the seismic design of a slope reinforced by a pile-anchor system is a complex and systematic work. The stability of the slope during earthquake activity is not completely unified with the safety of the pile-anchor structure. For example, when the slope is in the most stable state, the reinforced structure may already be in an unstable state^28^.Consequently, it is not appropriate to analyze the force and deformation of the pile-anchor structure and the seismic stability of the slope in isolation. In other words, the seismic design of reinforced slopes should be a multi-objective optimization problem^29^, and the force and deformation of the pile-anchor structure and the slope as well as their dynamic response must be considered.

Therefore, under the premise of considering the stability of the slope, the safety of the pile-anchor structure, and the dynamic response of the slope, this study proposes an optimization evaluation model based on multi-objective optimization (hereinafter referred to as the “*MOE* model”) to evaluate the seismic performance of a slope reinforced by a pile-anchor system, aiming to provide a new approach for the seismic reinforcement design of slopes. Specifically, the model selects three types of key physical quantities to construct a comprehensive optimization index system: slope stability indexes (displacement, acceleration), anti-slide pile indexes (displacement, bending moment, shear force), and anchor indexes (axial force and displacement). The comprehensive weights of each index are subsequently determined by the analytic hierarchy process (*AHP*) method and coefficient of variation (*COV*) method to comprehensively evaluate the seismic performance of slopes reinforced by the pile-anchor system. Finally, a simplified three-dimensional numerical model of the pile-anchor system coupled with the slope was established according to a prototype slope in Ludian County, Yunnan Province. The safety of the pile-anchor system itself and the stability of the slope during earthquake activity were analyzed for different working conditions.

## Design of multi-objective optimization evaluation model for the seismic performance of slopes reinforced by a pile-anchor system

**Figure 1:**
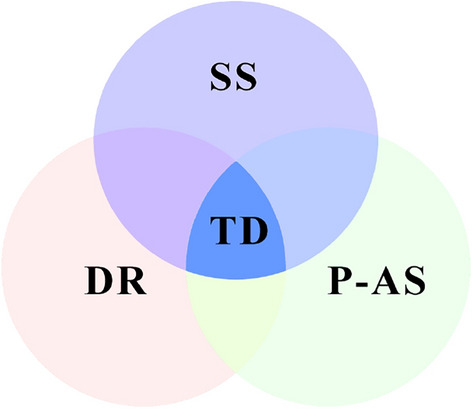
Conceptual diagram of the *MOE* model for the seismic performance of slopes reinforced by a pile-anchor system.

The essence of the proposed *MOE* model for measuring the seismic performance of slopes reinforced by a pile-anchor system is to seek the optimal target scheme (i.e., Target Design, *TD*) that can ensure the safety of the pile-anchor system (i.e., Pile-Anchor Safety, *P-AS*) and can simultaneously consider the dynamic response of the slope (i.e., Dynamic Response, *DR*) under the premise of meeting the stability of the slope (i.e., Slope Stability, *SS*) (Fig. 1).

### Determination of the optimization evaluation index

The design of slopes reinforced by a pile-anchor system in strong earthquake-prone areas must comprehensively consider the stability of the slope, the safety of the pile-anchor system and the dynamic response characteristics of slopes. Ensuring slope stability is the foremost consideration in the reinforcement design, and the mean displacement of the slope (*DIS*) can provide a more reliable indicator of slope stability^30,31^; therefore, the *DIS* is adopted as an evaluation index in the *MOE* model. The safety of the pile-anchor system is mainly determined by its force and deformation, while the pile displacement (*PD*), pile bending moment (*BM*), pile shear force (*SF*), anchor axial force (*AF*), and anchor displacement (*AD*) are the intuitive embodiments of its force and deformation characteristics. Therefore, the safety of the pile-anchor structure can be incorporated into the *MOE* model through the above five indexes. Moreover, the dynamic response of the slope can be characterized by the acceleration amplification factor (AAF, i.e., the ratio of peak ground acceleration measured in the slope to that observed at the slope foot). In summary, the *MOE* model proposed in this study for measuring the seismic performance of a slope reinforced by a pile-anchor system selects seven physical quantities as optimization evaluation indexes—namely *DIS*, *PD*, *BM*, *SF*, *AF*, *AD* and *AAF*.

### Construction of the index function

According to the relationship between the index value and optimization results, the indexes can be divided into three types: positive indexes that are positively correlated with the optimization results, negative indexes that are negatively correlated with the optimization results, and intermediate indexes whose optimization results are best when their values are closer to the intermediate value. In the present study, these three types of indexes correspond to three different normalization methods, as Eqs. (1)–(3). Based on this, the abovementioned seven indexes can be classified. The smaller the *DIS* and *AAF* values, the more stable is the slope; the smaller the *PD* value, the safer is the anti-slide pile; and finally, the smaller the *AD* value, the safer is the anchor. Therefore, these four indexes can be regarded as negative indexes, that are normalized using Eq. (2). In a practical project, the main failure pattern of the anti-slide pile is toppling deformation, and the phenomenon of being cut off and bent off rarely occurs; additionally, the main failure pattern of the anchor is the subsidence of the anchor head due to excessive displacement, while the situation of being pulled off rarely occurs^32^. In other words, *SF*, *BM*, and *AF* have large design tolerances. To give full play to the reinforcing effect of the pile-anchor system and take into account the system’s own safety, in this study, these three indexes are regarded as intermediate indexes that will be normalized by Eq. (3).

Through the actual monitoring data or numerical simulation results of the slope case reinforced by the pile-anchor system, the seven selected optimization evaluation indexes in "Determination of the optimization evaluation index" section—namely *DIS*, *PD*, *BM*, SF, *AF*, *AD*, and *AAF*, can be extracted and further normalized by the maximum–minimum normalization method to obtain the fuzzy matrix corresponding to the index system.

Assume that the number of schemes is *m*, and the number of optimization evaluation indexes for each scheme is *n.*

For a positive index, the normalization equation is as follows:

1 $$r(i,j) = A + B \cdot e^{{\frac{x(i,j) - x(i,j)\max }{{x(i,j)\max - x(i,j)\min }}}}$$

For a negative index, the normalization equation is as follows:

2 $$r_{(i,j)} = A + B \cdot e^{{\frac{{x_{(i,j)\min } - x_{(i,j)} }}{{x_{(i,j)\max } - x_{(i,j)\min } }}}}$$

For an intermediate index, the normalization equation is as follows:

3 $$r_{(i,j)} = \left\{ {\begin{array}{*{20}l} {A + B \cdot e^{{\frac{{x_{(i,j)} - x_{{(i,j){\text{mid}}}} }}{{x_{{(i,j){\text{mid}}}} - x_{(i,j)\min } }}}} ,x_{(i,j)\min } \le x_{(i,j)} \le x_{{(i,j){\text{mid}}}} } \hfill \\ {A + B \cdot e^{{\frac{{x_{{(i,j){\text{mid}}}} - x_{(i,j)} }}{{x_{{(i,j){\text{mid}}}} - x_{(i,j)\min } }}}} ,x_{{(i,j){\text{mid}}}} \le x_{(i,j)} \le x_{(i,j)\max } } \hfill \\ \end{array} } \right.$$

where *A* and *B* are constants and *A* + *B* = 100; in this study, we take *A* = 60 and *B* = 40; $x_{(i,j)\min }$,$x_{(i,j)\max }$, and $x_{{(i,j){\text{mid}}}}$ are the minimum, maximum, and median of the *i*th optimization index in the *j*th scheme, respectively; $r_{(i,j)}$ is the normalized value of the optimization evaluation index, that is, the relative membership degree. Accordingly, the fuzzy matrix can be determined as follows:

4 $$R = \left( {\begin{array}{*{20}l} {r(1,1)} & \ldots & {r(1,m)} \\ \vdots & \ddots & \vdots \\ {r(n,1)} & \cdots & {r(n,m)} \\ \end{array} } \right)$$

### Determination of the comprehensive weight of the optimization index

The comprehensive weight of the optimization index is determined by the combination of subjective and objective weights. The AHP method is used to determine subjective weights, and the *COV* method is used to determine objective weights. The equation to calculate the objective weight determined by using the *COV* method is:

5 $$wki = \frac{vi}{{\sum\nolimits_{i = 1}^{n} {vi} }}$$

where $vi$ is the *COV* of the *i*th optimization index.

After determining the subjective and objective weights, the comprehensive weight of each optimization index is obtained by employing the following equation:

6 $$w_{(i)} = \frac{{w_{{{\text{z}}i}} \cdot w_{ki} }}{{\sum\nolimits_{i = 1}^{n} {w_{zi} \cdot w_{ki} } }}$$

where $w_{zi}$ and $w_{ki}$ represent subjective weight and objective weight respectively.

### Calculation of the results of the optimization evaluation

By multiplying the fuzzy matrix and weight vector determined in "Construction of the index function" and "Determination of the comprehensive weight of the optimization index" sections, the corresponding fuzzy comprehensive evaluation value *k*(*j*) can be obtained by using Eq. (7). A larger *k*(*j*) value indicates a more effective reinforcement scheme.

7 $$k_{(j)} = \sum\limits_{i = 1}^{n} {\sum\limits_{j = 1}^{m} {w_{(i)} } } \cdot r_{(i,j)}$$

### Workflow of the *MOE* model

**Figure 2:**
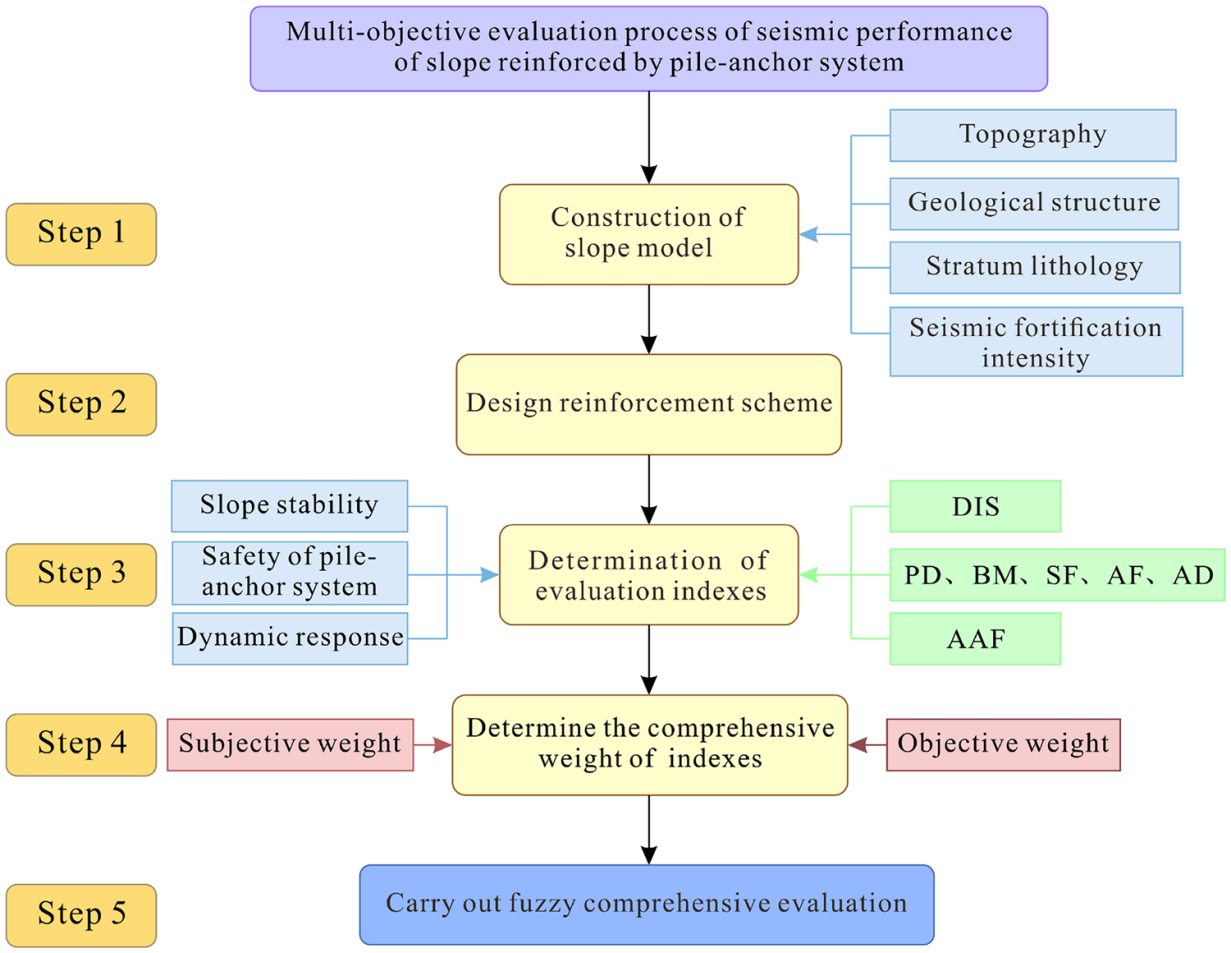
Workflow of the *MOE* model for evaluating the seismic performance of slopes reinforced by a pile-anchor system.

The specific workflow of the *MOE* model is divided into the following five steps (Fig. 2):

*Step 1* Construction of the slope model: In this step, it is necessary to collect detailed information about the topography, geological structure, stratigraphic lithology, and seismic precautionary intensity of the slopes reinforced by a pile-anchor structure. Additionally, external factors such as engineering disturbances in the vicinity of slopes (e.g., train moving load, reservoir water fluctuation, etc.) should also be identified. Subsequently, combined with satellite remote sensing data or UAV DEM data, a three-dimensional numerical model of slope can be established. For some simple slopes, a simplified numerical model can be established (e.g., a three-dimensional simplified model is established in FLAC3D in this study).

*Step 2* Designing of reinforcement schemes: In this step, the specific needs of the owner or customer, such as safety level, slope angle, and project budget, should be clarified. On this basis, by adjusting key parameters such as pile position, pile length, anchor length, anchor angle, and anchor row spacing, a table illustrating the pile-anchor reinforcement scheme is designed. Additionally, the corresponding numerical model of the slope reinforced by the pile-anchor system is established to perform numerical simulation.

*Step 3* Determination of the evaluation indexes: The selection of the optimization evaluation index is critical because there is a need to not only consider the stability of the slope but also to consider the safety of the pile-anchor system and the dynamic response characteristics of slopes. In the present study the mean displacement (*DIS*) and acceleration amplification factor (*AAF*) are selected as evaluation indexes to characterize the slope stability and dynamic response; additionally, pile displacement (*PD*), pile bending moment (*BM*), pile shear force (*SF*), anchor axial force (*AF*), and anchor displacement (*AD*) are selected as evaluation indexes to characterize the safety of the pile-anchor system. In other words, the *MOE* model includes seven evaluation indexes.

*Step 4* Determining the comprehensive weight of the indexes: First, the data of the seven evaluation indexes in Step 3 should be extracted; subsequently, the data should be normalized through the maximum-minimum normalization method. Successively, subjective weights and objective weights can be determined by the *AHP* method and the *COV* method, respectively. Finally, the combination of subjective weights and objective weights can be used to obtain the comprehensive weights of each evaluation index.

*Step 5* Conducting fuzzy comprehensive evaluation. The comprehensive optimization value of each reinforcement scheme can be determined by calculating the relative membership degree under different reinforcement schemes and the comprehensive weight of each evaluation index. A higher fuzzy comprehensive evaluation value indicates a more reasonable reinforcement scheme.

## Numerical reinforcement schemes

### Establishment of the numerical model

**Figure 3:**
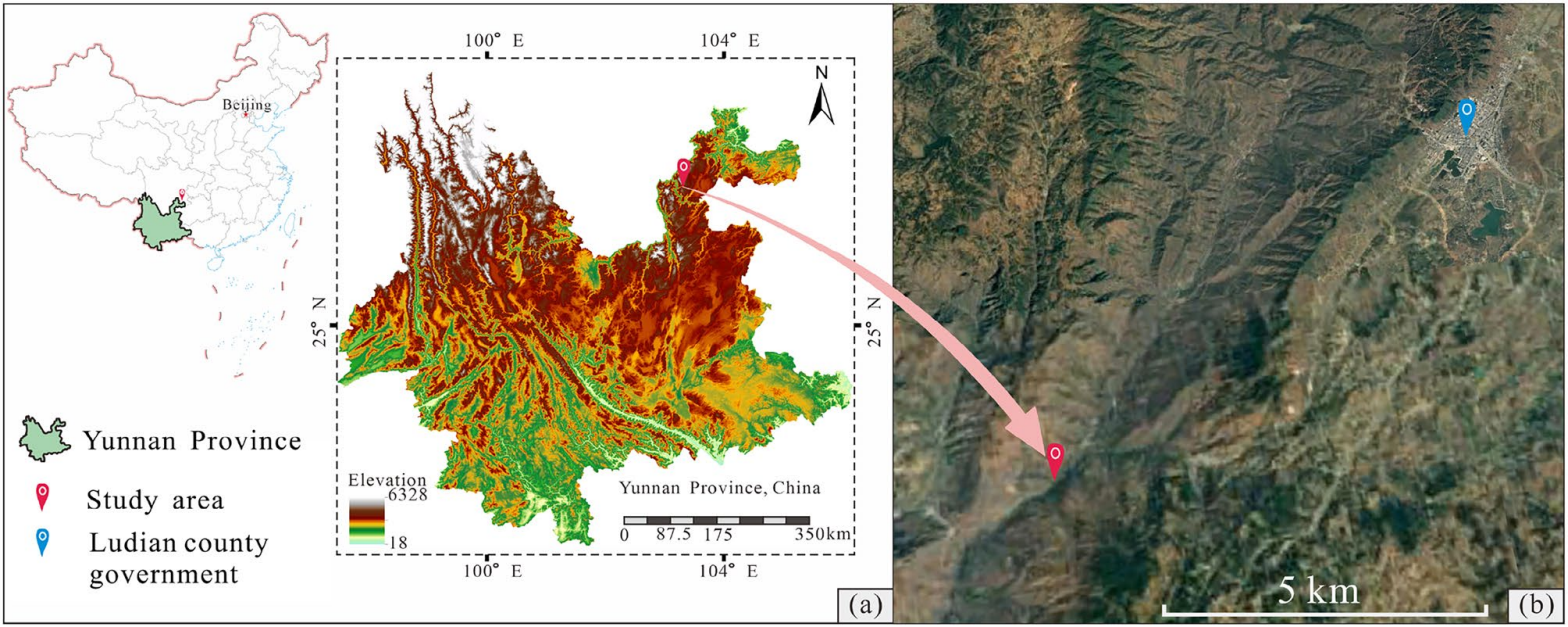
Location of the study area. (The map was generated by QGIS 3.28.1, https://qgis.org/en/site/).

**Figure 4:**
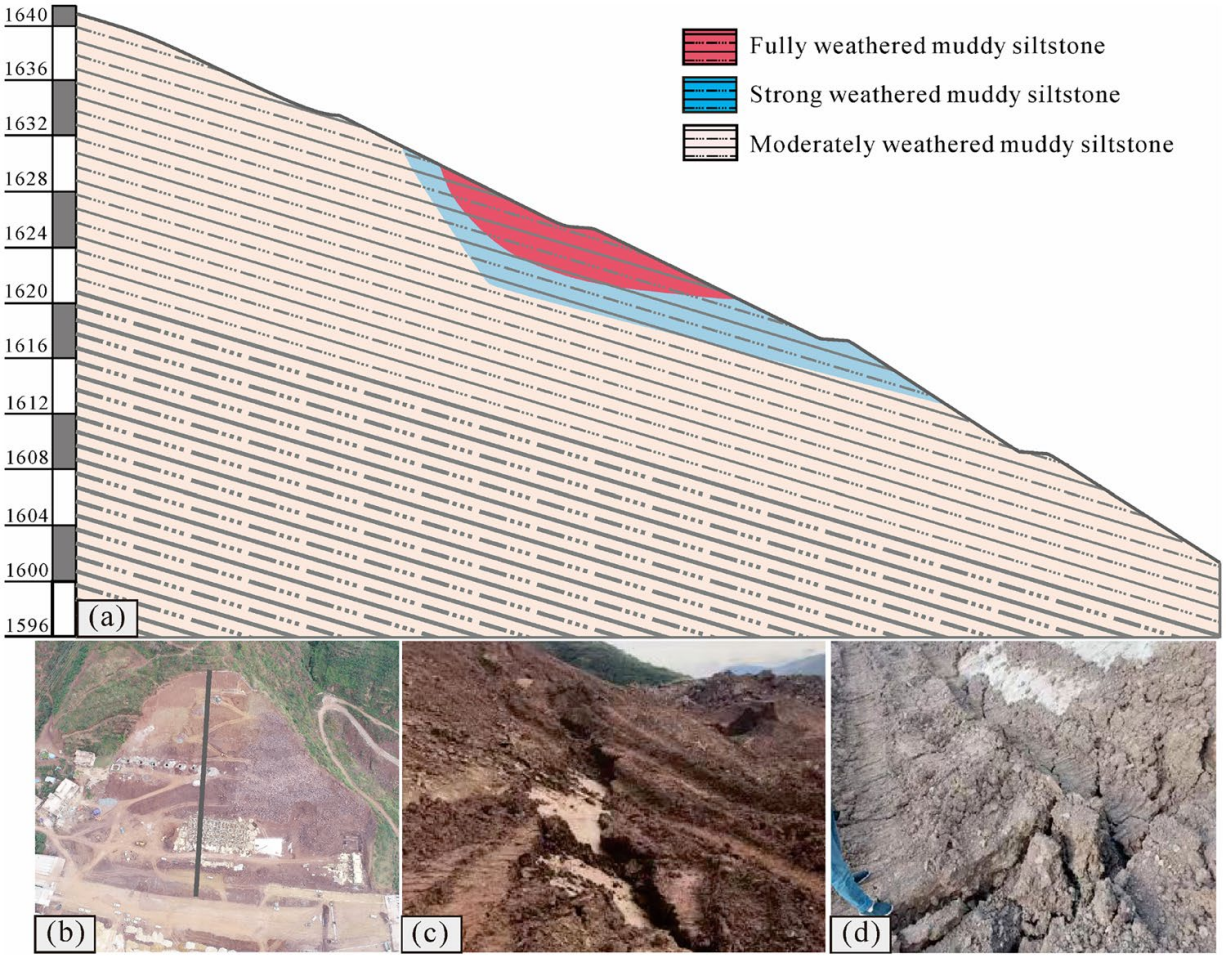
Geomorphology and engineering geological profile of the slope: (**a**) engineering geological profile; (**b**) geomorphology; (**c**) tensile cracks at the rear edge of the slope; and (**d**) flank shear cracks of the slope.

The study area is located in Ludian County, Yunnan Province, southwest China, near the Xiaojiang fault zone, which is characterized by a complex geological structure and frequent earthquakes, as shown in Fig. 3. On August 3, 2014, the Ludian earthquake with a magnitude of 6.5, struck this area, triggering a widespread occurrence of landslides that resulted in heavy casualties and property losses^33–35^. The geological profile and landform of the prototype slope are illustrated in Fig. 4. Its elevation is between 1610 and 1630 m, the slope is approximately 40°, and the slope volume is approximately 8000 m^3^. Based on on-site investigation, it has been determined that this prototype slope consists of alternating soft and hard rock layers, with its stability being controlled by the weak interlayer located between adjacent layers that have a dip angle of 15–20°.

**Figure 5:**
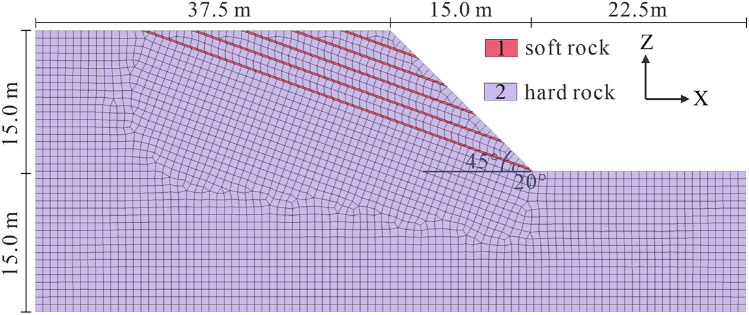
Numerical model of the slope.

The numerical model has a great influence on the simulation results^36–38^, and we make appropriate simplifications to construct the numerical model on the basis of the above prototype slope. The bedding slope angle is 45°, the rock dip angle is 20°, five weak interlayers with a thickness of 0.2 m are set in the upper part, and the bottom is bedrock. In this section, the spatial meshing element size, *Δl*, adheres to the rule proposed by Kuhlemeyer and Lysmer^39^ to guarantee the accuracy of the simulation. It is characterized as follows:

8 $$\Delta l \le \frac{\lambda }{10}{\text{to}}\frac{\lambda }{8}$$

where *Δl* is the meshing element size, and *λ* denotes the wavelength corresponding to the highest component frequency of the input wave. Hence, we set the grid size to 0.8 m, thus ensuring the complete propagation of seismic waves in the medium. Note that the meshing refinement should be discontinued after checking that the results do not change^40–42^. In summary, 18,630 nodes are generated, and 15,000 grid cells are delineated in the current model (Fig. 5). The physical and mechanical parameters of rock and soil mass are determined through field and laboratory tests (Table 1), and the Mohr–Coulomb strength criterion isused^43^. To minimize the influence triggered by boundary effects and ensure calculation accuracy under dynamic conditions, it is necessary to extend the model by implementing the following specifications^44,45^: (1) the distance from the foot of the slope to the right boundary is 1.5 times the slope height; (2) the distance from the top of the slope to the bottom boundary of the model is 2 times the slope height; and (3) The distance from the top of the slope to the left boundary is 2.5 times the slope height.

**Table 1:** Physical and mechanical parameters of the slope.

	Young modulus *E* (MPa)	Poisson ratio	Unit weight *γ* (kN/m^3^)	Cohesion *c* (kPa)	Friction angle *φ* (°)
Soft rock	200	0.33	1720	3.6	20
Hard rock	840	0.25	2300	70	35

### Numerical simulation conditions

**Figure 6:**
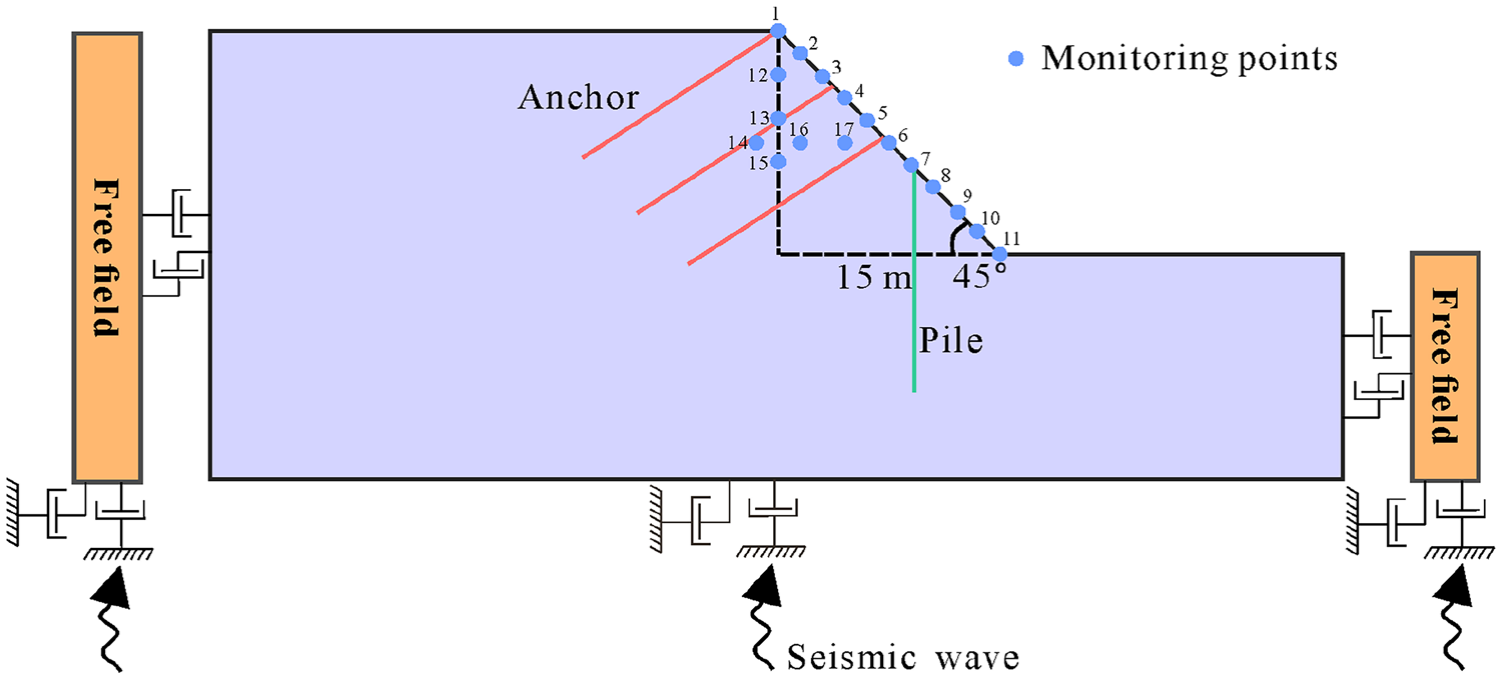
Diagram of boundary conditions of the numerical model.

**Figure 7:**
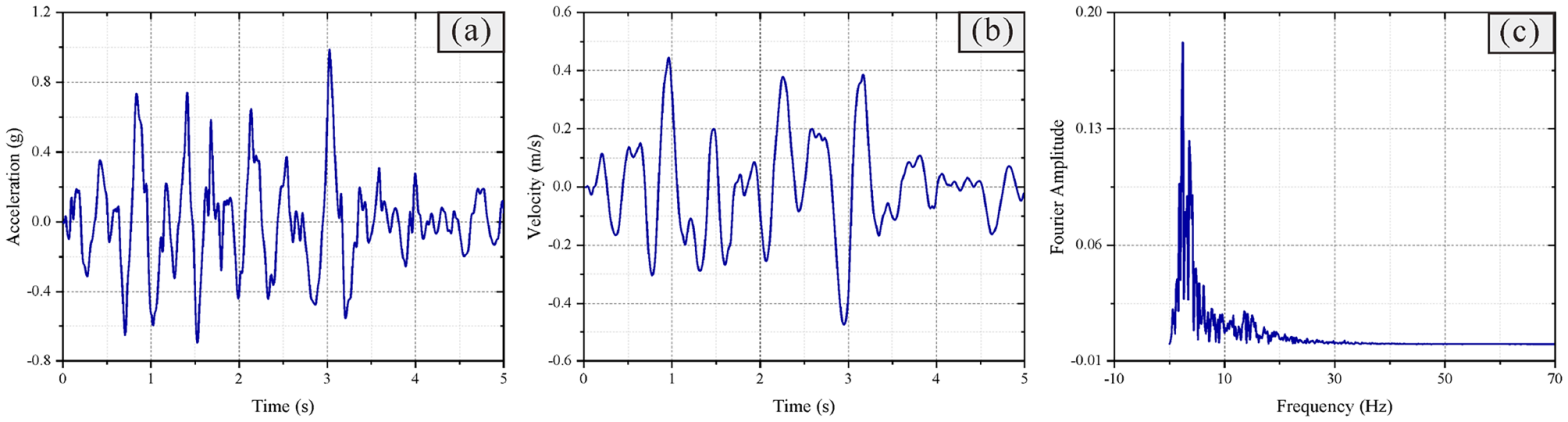
Seismic wave data.

Boundary conditions must be considered in the simulation of seismic conditions^46^, a free-field boundary around the model can better simulate the semi-infinite space, and the static boundary (viscous boundary) at the bottom can solve the problem of reflection of seismic waves^47,48^. The side boundary of the grid is coupled with the free field boundary through the damper to ensure that the input seismic wave is not distorted (Fig. 6), local damping is adopted in this dynamic analysis, and the damping coefficient is 0.157^48,49^. The seismic wave adopts the larger peak part of the real wave of 2008 Wenchuan earthquake that occurred in Sichuan (Fig. 7).

**Figure 8:**
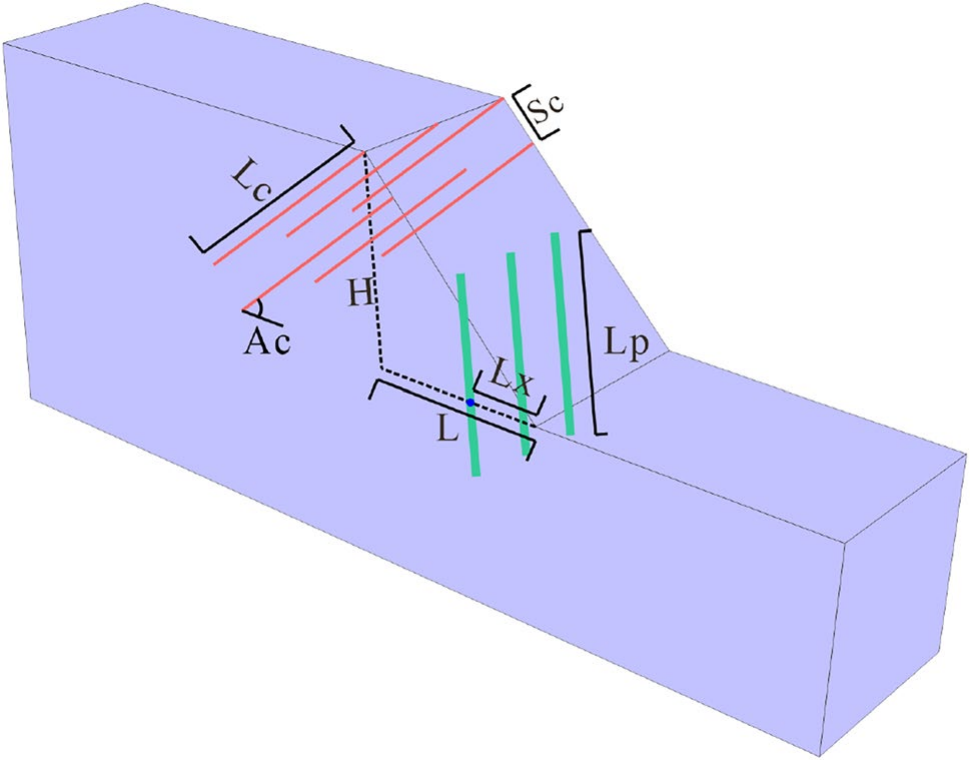
Numerical model diagram of the slope reinforced by the pile-anchor system, where *Lx* is the distance from the slope foot to the anti-slide pile; *Lp* is the pile length; *Lc* is the anchor length; *Ac* is the anchor angle; and *Sc* is the anchor row spacing.

Considering that the layout of the pile-anchor system greatly influences its reinforcement effect, 30 groups of simulated working conditions are established in this study through the control variable method for the following five variables: pile position, pile length, anchor length, anchor angle and anchor row spacing (Fig. 8 and Table 2). Moreover, it should be noted that both anti-slide piles and anchors adopt the structural units embedded in FLAC3D, and their physical and mechanical parameters are presented in Tables 3 and 4.

**Table 2:** Numerical simulation schemes.

Scheme	*Lx*/*L*	*Lp* (m)	*Lc* (m)	*Ac* (°)	*Sc* (m)
1–6	0.0,0.2,0.4,0.6,0.8,1.0	14	18	15	3
7–12	0.4	5,8,11,14,17,20	18	15	3
13–18	0.4	14	9,12,15,18,21,24	15	3
19–24	0.4	14	18	3,9,15,21,27,33	3
25–30	0.4	14	18	15	1,2,3,4,5,6

**Table 3:** Physical and mechanical parameters of the anti-slide piles.

Parameter	Value	Parameter	Value	Parameter	Value
Young modulus	30 GPa	Coupling-cohesion-shear	1.9 × 10^7^ Pa	Coupling-cohesion-normal	1.9 × 10^7^ Pa
Poisson ratio	0.20	Coupling-stiffness-shear	1.0 × 10^11^ N/m^2^	Coupling-stiffness-normal	1.0 × 10^11^ N/m^2^
Moi-z	2.0 m^4^	Coupling-stiffness-shear	23°	Coupling-friction-normal	23°
Moi-y	4.5 m^4^	Density	2500 kg/m^3^	Coupling-gap-normal	on
Moi-polar	6.5 m^4^	Cross-sectional-area	0.25m^2^	Perimeter	2.0 m

**Table 4:** Physical and mechanical parameters of the anchor.

Elastic modulus (GPa)	Density (g/cm^3^)	Cross-sectional (m^2^)	Grount-cohesion (N/m)	Grount-perimeter (m)
20	7800	7.065e−4	3.5e6	0.314

## Analysis of the seismic reinforcement effect of the pile-anchor system

### Influence of the pile position on the reinforcement effect

**Figure 9:**
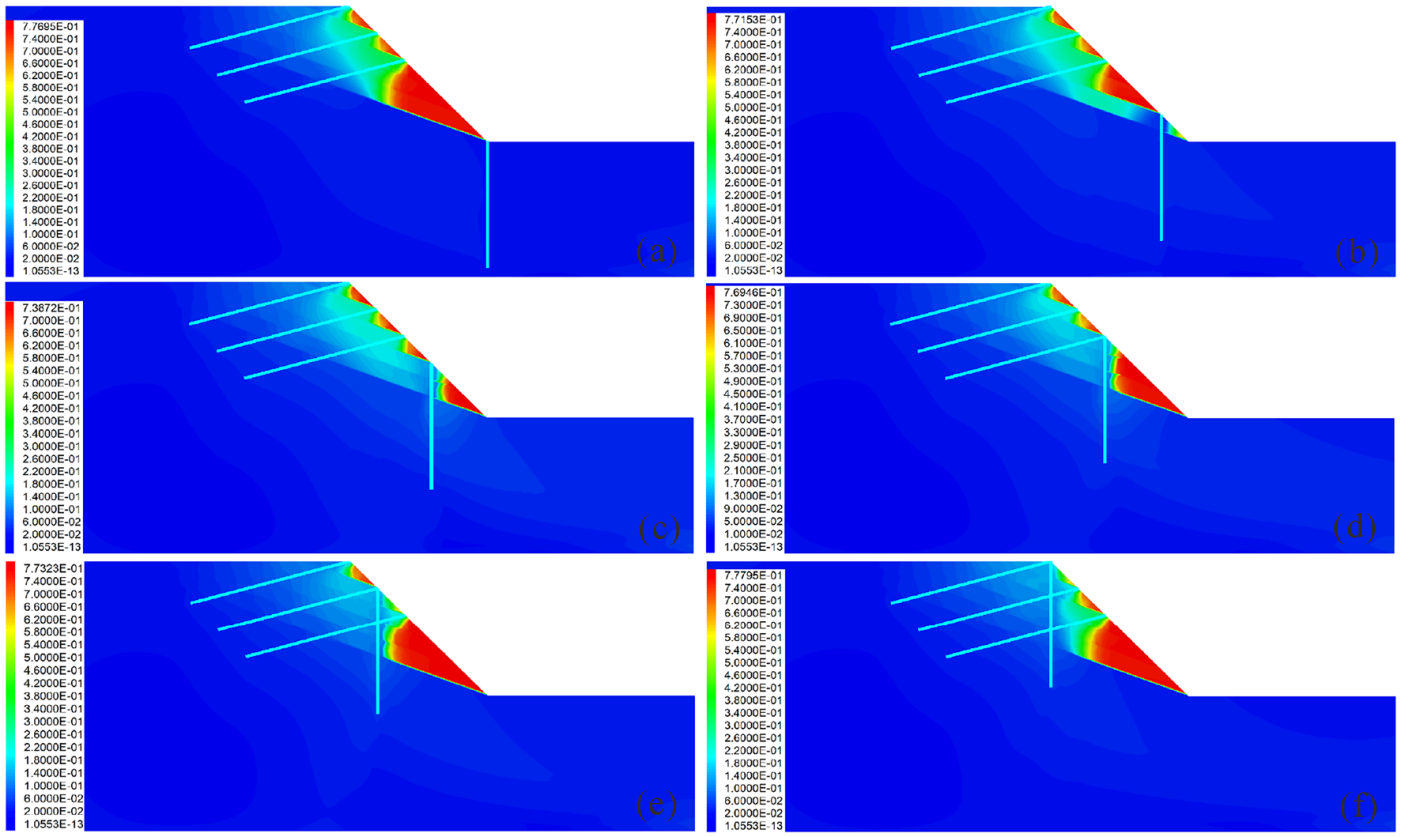
Displacement cloud diagram under different pile positions: (**a**) *Lx/L* = 0.0; (**b**) *Lx/L* = 0.2; (**c**) *Lx/L* = 0.4; (**d**) *Lx/L* = 0.6; (**e**) *Lx/L* = 0.8; and (**f**) *Lx/L* = 1.0.

**Figure 10:**
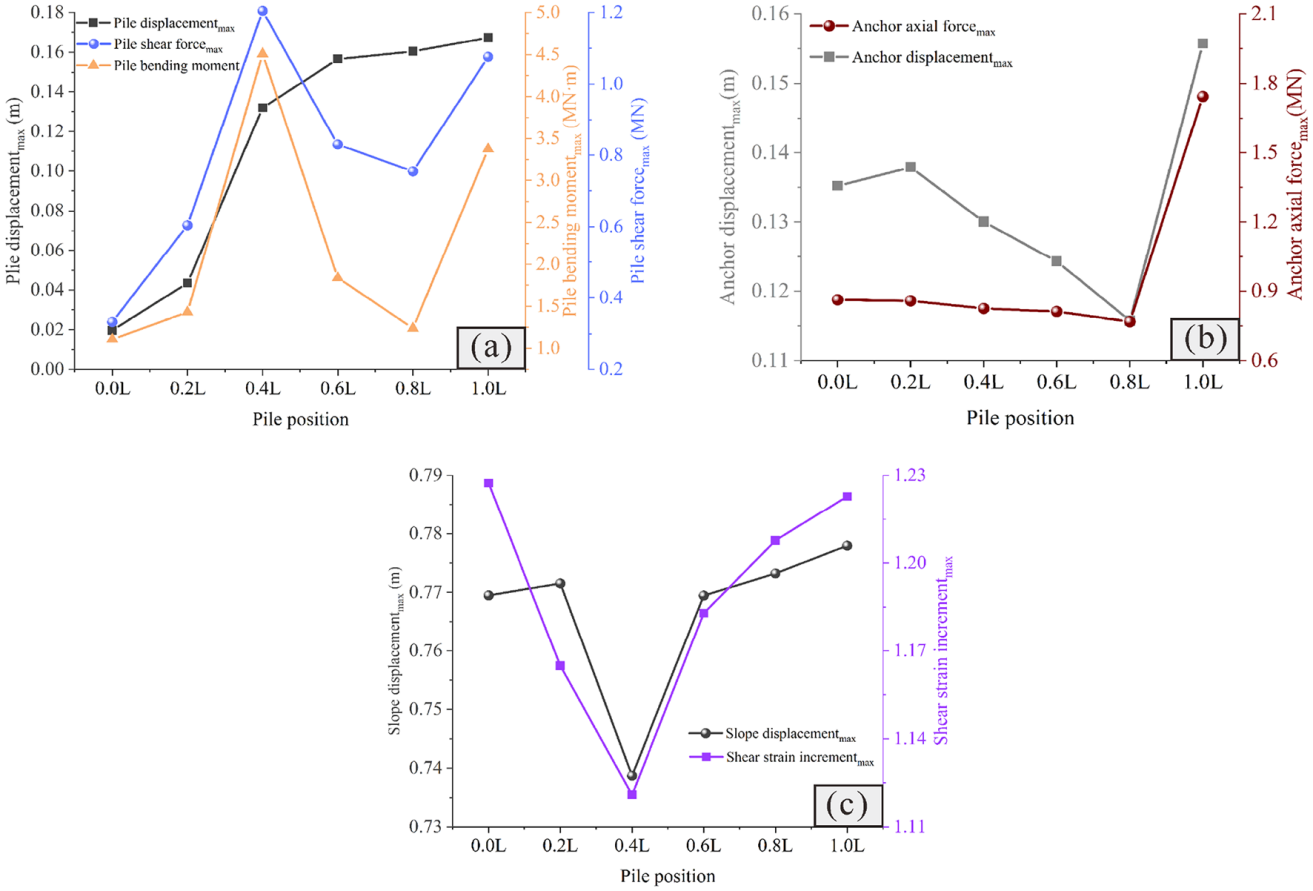
Comparison of the deformation and force of the pile-anchor structure and slope under different pile positions: (**a**) Displacement, bending moment and shear force of the anti-slide pile; (**b**) Axial force and displacement of the anchor; (**c**) Maximum displacement and maximum shear strain increment of the slope.

The analysis shows that the pile position has a highly discernible influence on the reinforcement effect (Figs. 9 and 10). Specifically, when the pile is arranged at the foot of the slope (Fig. 9a), the pile experiences minimal displacement, bending moment and shear force (Fig. 10a), whereas the slope undergoes significant displacement and shear strain, and the anchor encounters large axial force and displacement (Fig. 10b and c). This indicates that the anti-slide pile does not fully fulfill its supporting function at this time; this results in an increasing sliding force borne by the anchor with a higher risk. When the pile is arranged at the top of the slope (Fig. 9f), the displacement, bending moment and shear force of the pile are large (Fig. 10a); the deformation of the anchor is severe (Fig. 10b); and the slope body is subjected to serious shear action, resulting in the largest slope displacement and the worst reinforcement effect (Fig. 10c). When piles are arranged in the middle and upper part of the slope (Fig. 9d and e), the lower part of the slope is prone to large deformation due to the lack of support; thus, the reinforcement effect is not satisfactory. When piles are arranged in the middle and lower part of the slope (Fig. 9b and c), the deformation of the slope is smaller and the reinforcement effect is good. Specifically, when *Lx/L* = 0.4 (Fig. 9c), the reinforcement effect is best and the deep sliding surface is divided into several secondary shallow sliding surfaces. However, when in this condition, the pile displacement, bending moment and shear force are slightly larger, and this needs to be given due attention.

### Influence of pile length on the reinforcement effect

**Figure 11:**
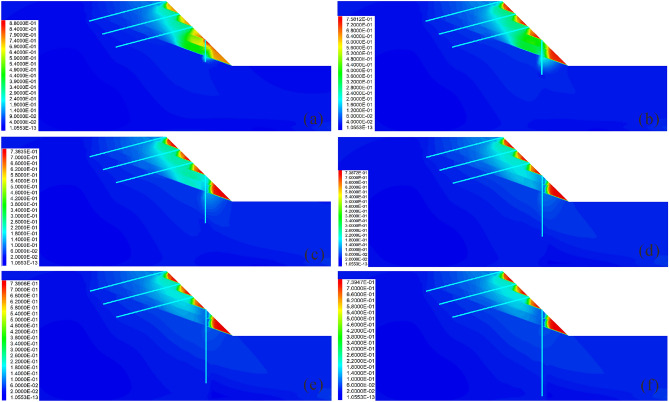
Displacement cloud diagram under different pile lengths: (**a**) *Lp* = 5 m; (**b**) *Lp* = 8 m; (**c**) *Lp* = 11 m; (**d**) *Lp* = 14 m; (**e**) *Lp* = 17 m; (**f**) *Lp* = 20 m.

**Figure 12:**
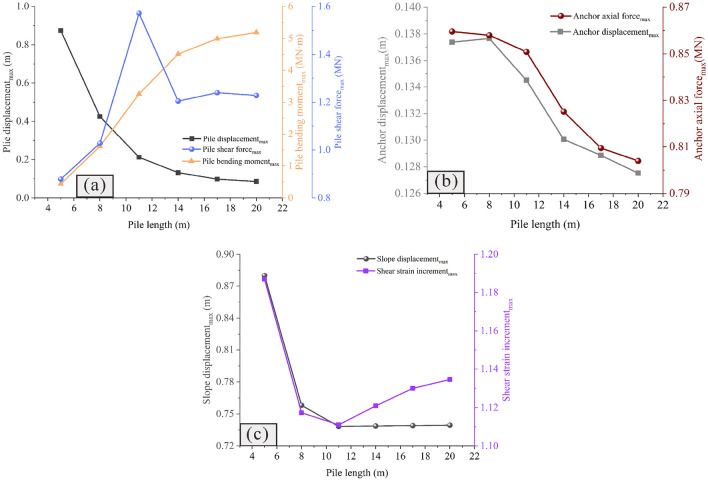
Comparison of the deformation and force of the pile-anchor structure and slope under different pile lengths: (**a**) Displacement, bending moment and shear force of the anti-slide pile; (**b**) Axial force and displacement of the anchor; (**c**) Maximum displacement and maximum shear strain increment of the slope.

Figures 11 and 12 show the influence of the pile length on the reinforcement effect. It is observed that when the pile length is short, the slope usually has a large displacement (Fig. 11a–c), and the displacement of the anti-slide pile is extremely large, with the risk of tipping (Fig. 12a).Additionally, as the pile length increases, the sliding force of the slope borne by the anti-slide pile increases, and the anchor axial force and displacement decrease continuously, gradually becoming safe (Fig. 12b). It is important to note that once the pile length exceeds 11 m, further increasing its length does not significantly affect the slope displacement; however, this can lead to an increase in the shear strain increment (Fig. 12c), indicating that there exists an optimal pile length for anti-slide piles.

### Influence of the anchor length on the reinforcement effect

**Figure 13:**
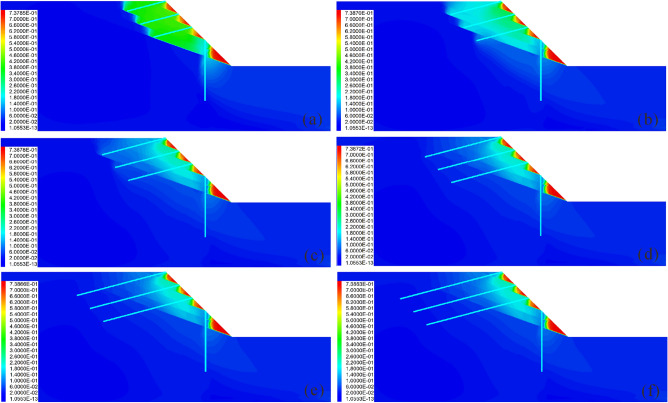
Displacement cloud diagram under different anchor lengths: (**a**) *Lc* = 9 m; (**b**) *Lc* = 12 m; (**c**) *Lc* = 15 m; (**d**) *Lc* = 18 m; (**e**) *Lc* = 21 m; (**f**) *Lc* = 24 m.

**Figure 14:**
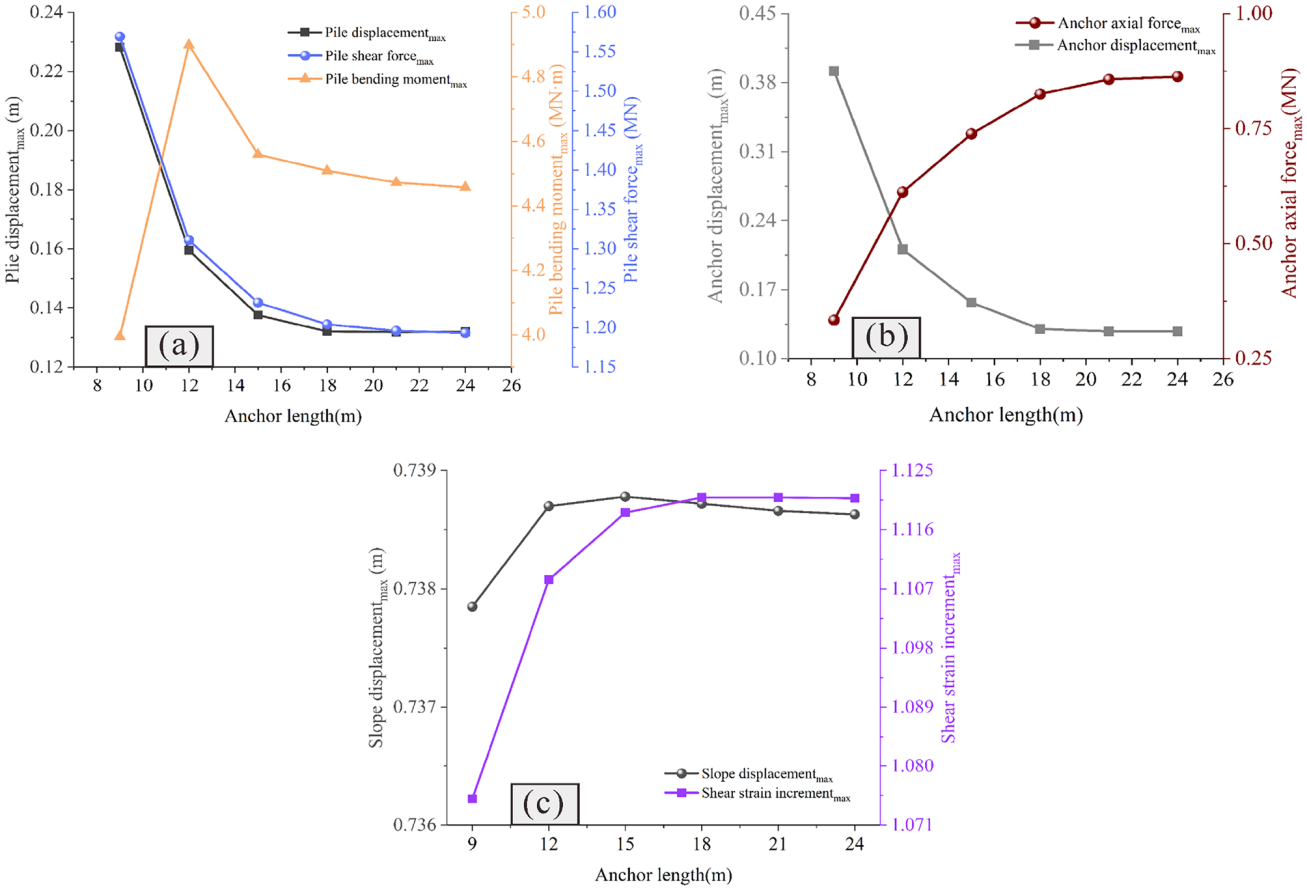
Comparison of the deformation and force of the pile-anchor structure and slope under different anchor lengths: (**a**) Displacement, bending moment and shear force of the anti-slide pile; (**b**) Axial force and displacement of the anchor; (**c**) Maximum displacement and maximum shear strain increment of the slope.

Figures 13 and 14 show the influence of the anchor length on the reinforcement effect. It is observed that when the anchor is short, the slope has the risk of overall sliding (Figs. 13a and b). With the increase of the anchor length, the displacement of the anchor decreases gradually, and the axial force increases continuously (Fig. 14b), thus indicating that when the anchor length is large, attention should be paid to increasing its tensile capacity. Similar to the pile length, when the anchor length reaches a certain value, the displacement and shear strain increment of the slope remain unchanged with increasing anchor length (Fig. 14c). This indicates that similar to the pile length, there exists an optimal value for the anchor length, and that increasing the anchor length beyond this value will not only fail to improve the reinforcement effect, but will also make the anchor bear greater tension and increase its risk.

### Influence of the anchor angle on the reinforcement effect

**Figure 15:**
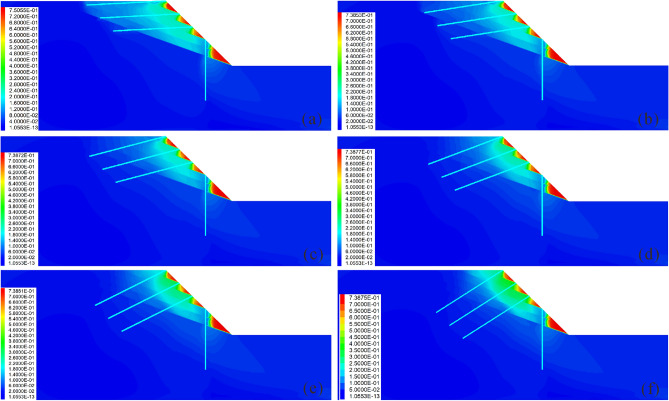
Displacement cloud diagram under different anchor angles: (**a**) *Ac* = 3°; (**b**) *Ac* = 9°; (**c**) *Ac* = 15°; (**d**) *Ac* = 21°; (**e**) *Ac* = 27°; (**f**) *Ac* = 33°.

**Figure 16:**
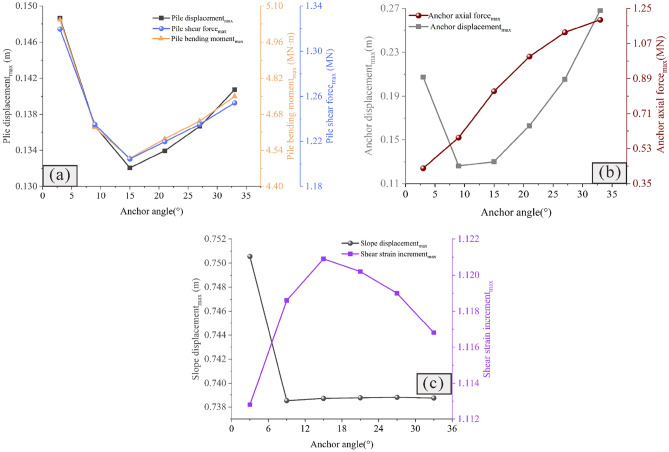
Comparison of the deformation and force of the pile-anchor structure and slope under different anchor angles: (**a**) Displacement, bending moment and shear force of the anti-slide pile; (**b**) Axial force and displacement of the anchor; (**c**) Maximum displacement and maximum shear strain increment of the slope.

Figures 15 and 16 show the influence of the anchor angle on the reinforcement effect. It is observed that when the anchor angle is extremely small (Fig. 15a), the displacement of the slope is larger (Fig. 16c); additionally, the displacement, bending moment and shear force of the pile are the largest at this anchor angle (Fig. 16a), indicating that the anti-slide pile is in the most dangerous state. At this time, the anchor axial force is the smallest (Fig. 16b), indicating that the anchor has not yet been fully used. Therefore, caution should be exercised to ensure that the anchor angle is not extremely small. When the anchor angle is extremely large, the anchor has a large axial force and displacement (Fig. 16b), indicating that the anchor is more dangerous. It should be noted that when the anchor angle is 15°, the bending moment, displacement, and shear force of the pile are the smallest (Fig. 16a); the displacement of both the slope and the anchor are also smaller (Figs. 16b and c), and the axial force of the anchor is in the middle level (Fig. 16b). At this time, the anchor can give full play to its reinforcement effect, and the anti-slide pile is also in the safest state. Therefore, in the case of the slope considered in this study, 15° is the optimal anchor angle.

### Influence of the anchor row spacing on the reinforcement effect

**Figure 17:**
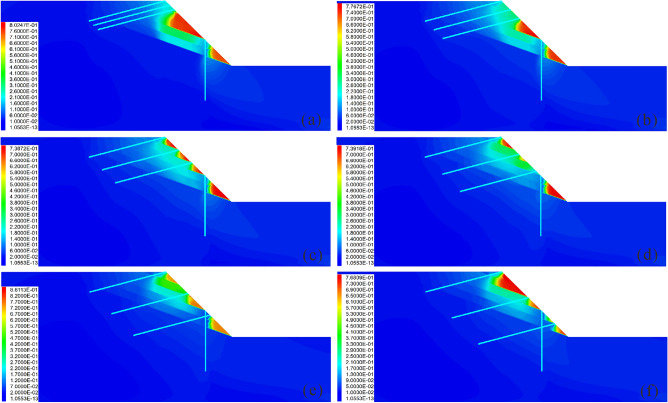
Displacement cloud diagram under different anchor row spacing values: (**a**) *Sc* = 1 m; (**b**) *Sc* = 2 m; (**c**) *Sc* = 3 m; (**d**) *Sc* = 4 m; (**e**) *Sc* = 5 m; (**f**) *Sc* = 6 m.

**Figure 18:**
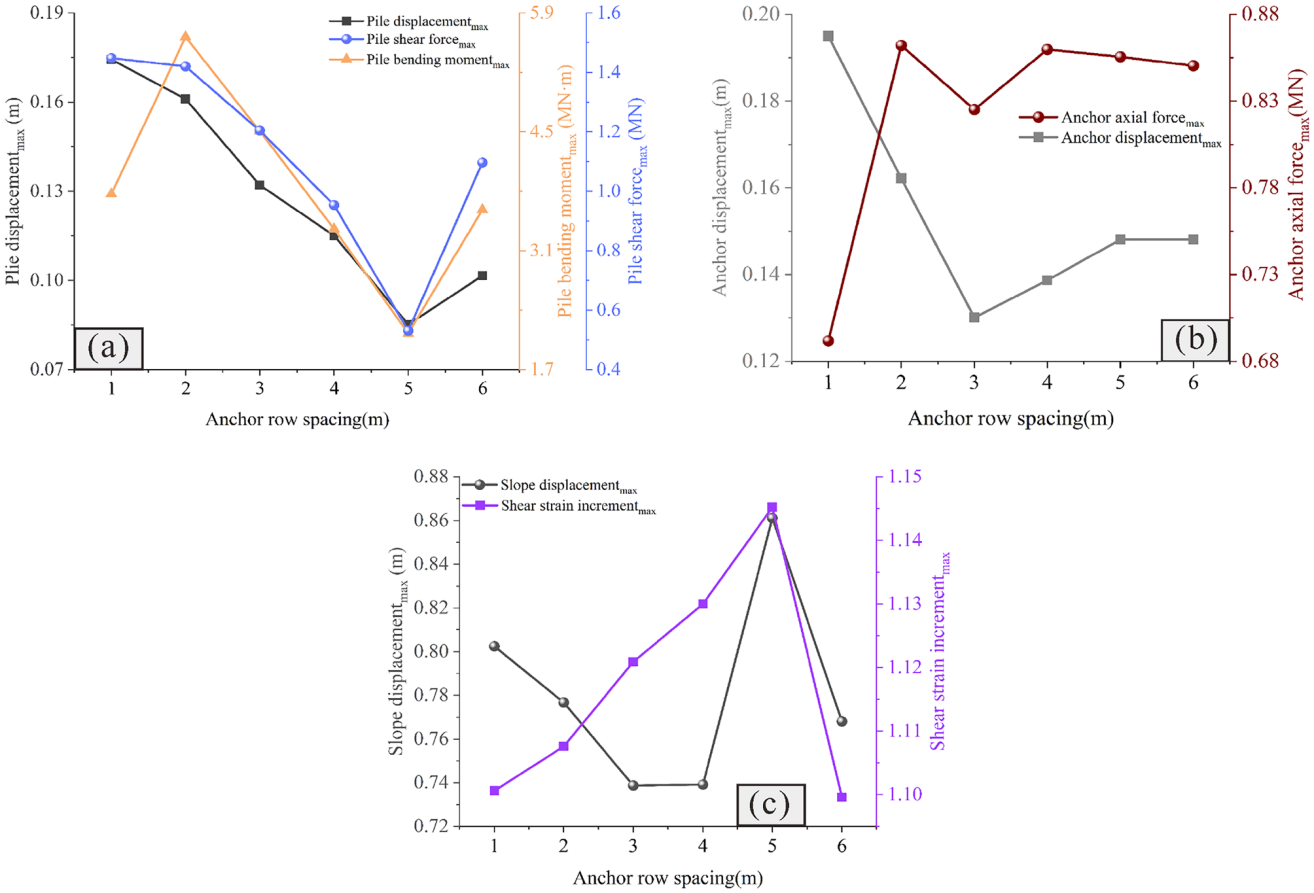
Comparison of the deformation and force of the pile-anchor structure and slope under different anchor row spacing values: (**a**) Displacement, bending moment and shear force of the anti-slide pile; (**b**) Axial force and displacement of the anchor; (**c**) Maximum displacement and maximum shear strain increment of slope.

Figures 17 and 18 show the influence of the anchor row spacing on the reinforcement effect. It is observed that when the anchor row spacing is extremely small, the reinforcement range is limited, and the unreinforced part is prone to large deformation (Fig. 17a and b). When the anchor row spacing is extremely large, due to the elevation amplification effect of ground motion in the earthquake, the upper part of the slope is prone to experiencing large deformation (Fig. 17f). Therefore, extremely small or extremely large anchor row spacing is not conducive to slope stability. With the anchor row spacing of 5 m, the slope is observed to be strongly sheared, consequently producing the maximum slope displacement (Fig. 18c). The minimum slope displacement is observed at the anchor row spacing of 3 m and 4 m (Fig. 18c). Further analysis shows that when the anchor row spacing is 4 m, the deformation of the upper part of the slope is less as compared to that when the anchor row spacing is 3 m (Fig. 17c, d); this indicates that the deformation of the slope is effectively limited. Additionally, when the row spacing is 4 m, the bending moment, displacement and shear force of the pile are all less as compared to their corresponding values when the anchor row spacing is 3 m (Fig. 18a). In summary, 4 m is the optimal anchor row spacing.

## Analysis of the evaluation results

### Determination of the index weight

According to Step 4 mentioned in "Workflow of the MOE model" section, the data of seven optimization evaluation indexes can be extracted through numerical simulation, and the corresponding comprehensive weight of each index can be calculated. To provide a more precise depiction of the overall slope deformation and dynamic response, the index *DIS* in this study refers to the average value of the maximum displacement of multiple monitoring points (e.g., monitoring points 1, 3, 5, 7, 9, 11–17 in Fig. 6) on the slope surface and within the slope; additionally, the index *AAF* refers to the maximum value of *AAF* at multiple monitoring points (e.g., monitoring points 1–11 in Fig. 6) on the slope surface.

**Figure 19:**
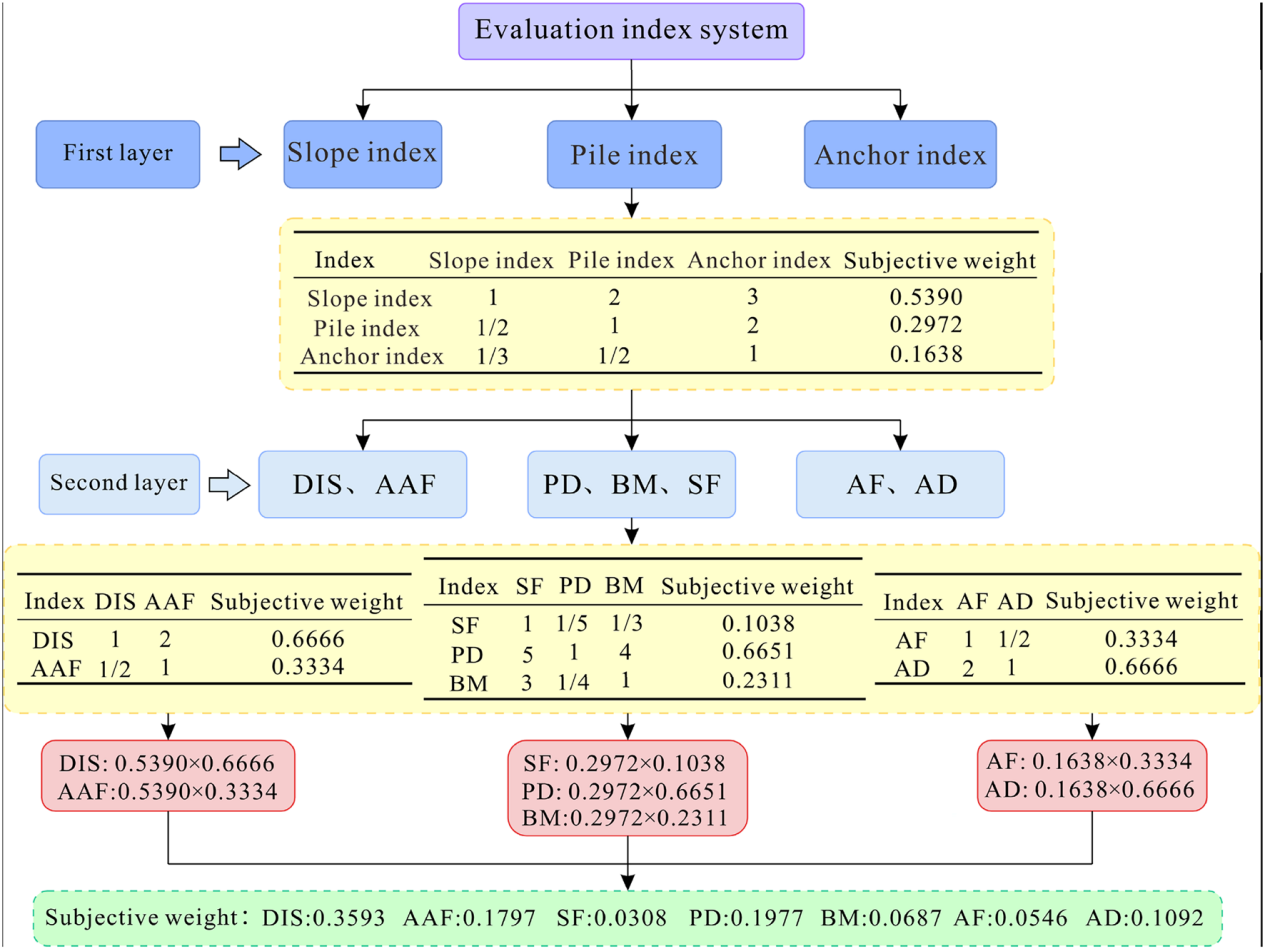
Schematic of determining subjective weights by using the *AHP* method.

In this study, the *AHP* method is employed to determine the subjective weight of each index, and the importance of each index is defined by the 1–9 scaling method, combined with the intention of decision-makers, the judgment of geological disaster experts and engineering experience. Note that the indexes need to be stratified as shown in Fig. 19. Specifically, the first layer is divided into three types: slope indexes (*DIS*, *AAF*), anti-slide pile indexes (*PD*, *BM*, *SF*), and anchor indexes (*AF*, *AD*). After determining the subjective weight of the three types of indexes in the first layer, the proportion of the weight of each index in the second layer can be further determined; finally, through the data collected in prior steps, the subjective weight of each optimization index can be obtained.

The judgment matrix of the first layer indexes is shown in Table 5. The maximum eigenvalue of the judgment matrix (${\lambda }_{max}$) is 3.009; the random index (RI) is 0.58; the consistency index (*CI*) is 0.0046; and the consistency ratio (*CR*) is 0.0079 and less than 0.1, thus meeting the consistency requirements. The judgment matrices of slope indexes (*DIS*, *AAF*) and anchor indexes (*AF*, *AD*) in the second layer are shown in Tables 6 and 7 respectively, both of which are second-order matrices that inevitably meet the consistency requirements. The judgment matrix of the anti-slide pile indexes (*PD*, *BM*, *SF*) in the second layer is shown in Table 8, where ${\lambda }_{max}$ is 3.087, *RI* is 0.58, *CI* is 0.0435, and *CR* is 0.0749 and less than 0.1, thus meeting the consistency requirements.

**Table 5:** The judgment matrix and subjective weight of the three types of indexes in the first layer.

Index	Slope index	Pile index	Anchor index	Subjective weight
Slope index	1	2	3	0.5390
Pile index	1/2	1	2	0.2972
Anchor index	1/3	1/2	1	0.1638

**Table 6:** The judgment matrix and subjective weight of slope indexes in the second layer.

Index	*DIS*	*AAF*	Subjective weight
*DIS*	1	2	0.6666
*AAF*	1/2	1	0.3334

**Table 7:** The judgment matrix and subjective weight of anchor indexes in the second layer.

Index	*AF*	*AD*	Subjective weight
*AF*	1	1/2	0.3334
*AD*	2	1	0.6666

**Table 8:** The judgment matrix and subjective weight of pile indexes in the second layer.

Index	*SF*	*PD*	*BM*	Subjective weight
*SF*	1	1/5	1/3	0.1038
*PD*	5	1	4	0.6651
*BM*	3	1/4	1	0.2311

Because conducting evaluation using only the *AHP* method is easily affected by personal subjective will and may lead to ignoring some important factors, this study adopts the *COV* method to calculate the objective weight of each index to compensate for the shortcomings of the *AHP* method. *COV* is a statistic that measures the dispersion degree of data and is defined as the ratio of the standard deviation to the mean of the data set^50^. As an objective weighting method, the *COV* method can determine the objective weight of each index through the *COV* of each index, that can objectively reflect the change in the index data^51,52^. The larger the *COV* of the index data, the greater is the degree of data dispersion, indicating that it is more difficult for this index to achieve the target value and should be given a larger weight, and vice versa. The process of assigning objective weight is as follows: first, we calculate the standard deviation *σ* and the mean value *μ* of the data of each index and obtain its *COV* according to Eq. (9). Subsequently, we normalize the *COV* and calculate the objective weights of each index through Eq. (5).

9 $$v_i = \frac{\sigma_i}{{\mu_i}}$$

After obtaining the subjective and objective weights through the *AHP* and *COV* methods, respectively, the comprehensive weight of each index can be determined by Eq. (6). Table 9 shows the values of different types for weights of each index.

**Table 9:** Different types of weights for each index.

Optimization indexes	Subjective weight	Objective weight	Comprehensive weight
*DIS*	0.3593	0.0671	0.1702
*AAF*	0.1797	0.0612	0.0777
*PD*	0.1977	0.3597	0.5019
*BM*	0.0687	0.1546	0.0750
*SF*	0.0308	0.1022	0.0223
*AF*	0.0546	0.1138	0.0439
*AD*	0.1092	0.1414	0.1090

### Comparison of the evaluation results

**Figure 20:**
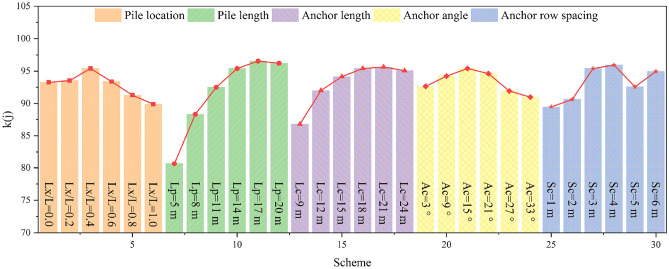
Evaluation results. From left to right, it corresponds to the fuzzy comprehensive evaluation values of the 30 numerical simulation schemes in Table 2. A large fuzzy comprehensive evaluation value indicates a good reinforcement effect.

The fuzzy comprehensive evaluation value can be calculated according to Eq. (7); this is displayed in Fig. 20. It is evident that the reinforcement effects of different numerical simulation schemes vary greatly.

Regarding the pile position, with the pile position gradually moving from the foot to the top of the slope, the reinforcement effect first increases and subsequently decreases. When *Lx/L* = 0.4, the reinforcement effect is the best, the slope stability is effectively improved, and the safety of the pile-anchor system is fully used. It should be noted that the reinforcement effect of the pile located in the middle and upper part of the slope is generally poor, which is inconsistent with the results of previous researches^53^. We attribute this to the fact that previous studies focused predominantly on the safety of the reinforcement structure itself, and did not fully consider the lack of support for the rock and soil mass in front of the pile when the pile is arranged in the middle and upper part of the slope. Additionally, as mentioned in "Influence of the pile position on the reinforcement effect" section, although the arrangement of piles at the foot of the slope can have a certain reinforcing effect, the supporting capacity of the pile-anchor system cannot be fully used in this position. Therefore, the commonly-used arrangement of piles at the foot of the slope in previous projects is not the optimal choice for achieving the best reinforcing effect.

Regardingthe anchor length, the effectiveness of the reinforcement is poor when the anchor length is short. Subsequently, with an increase in the anchor length, the reinforcement effect first increases and subsequently decreases; therefore, there exists an optimal anchor length.

In terms of the anchor angle, the reinforcement effect for the slope considered in this study is the best when the anchor angle is 15°; when angles increase beyond this value, the reinforcement effect subsequently decreases sharply. Therefore, the anchor angle cannot be extremely large in seismic reinforcement projects on slopes.

Regarding the anchor row spacing, when the row spacing is extremely small, the reinforcement range is highly limited. When the row spacing is significantly large, large deformation easily occurs between the anchors due to lack of reinforcement. Hence, extremely small or extremely large row spacing is not conducive for the seismic reinforcement of slopes. The reinforcement effects of anchor row spacing of both 3 m and 4 m 4 m do not differ widely; however, 4 m is observed to be the optimal anchor row spacing, which is highly consistent with the numerical simulation results of "Influence of the anchor row spacing on the reinforcement effect" section.

## Discussion

### Influence of the method of obtaining the displacement index on the evaluation effect

**Figure 21:**
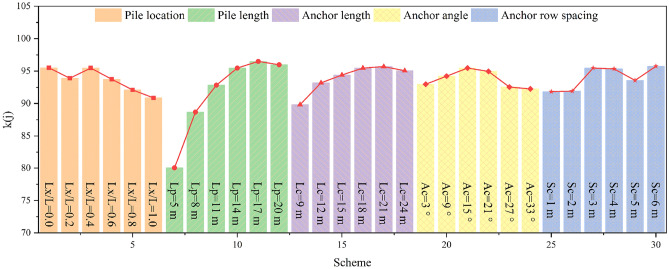
Evaluation results when the displacement index is *DIS*_*max*_. From left to right, it corresponds to the fuzzy comprehensive evaluation values of the 30 numerical simulation schemes in Table 2. A large fuzzy comprehensive evaluation value indicates a good reinforcement effect.

The displacement monitoring curve of the slope is often used to identify the evolution stage of the slope^46,54^, and is an important indicator of slope stability. Therefore, the selection of displacement monitoring points may have a certain impact on the identification results of slope evolution stage and slope stability. Consequently, to examine how the method of obtaining the displacement affects the evaluation result, this section conducts an assessment using the maximum displacement of the slope (*DIS*_*max*_) as the displacement index of the *MOE* model, and compares the new evaluation results with those of “Comparison of the evaluation results” section, in which the average displacement of the slope (*DIS*) is adopted. It should be noted that when *DIS*_*max*_ is used as the displacement index, the coefficient of variation of the displacement index data changes, resulting in changes in the objective weight and comprehensive weight of each index. The latest values of different types of weights for each index are presented in Table 10. The new evaluation results are displayed in Fig. 21, revealing some discrepancies with the results of "Comparison of the evaluation results" section.

**Table 10:** Different types of weights for each index when the displacement index adopted is *DIS*_*max*_.

Optimization indexes	Subjective weight	Objective weight	Comprehensive weight
*DIS*_*max*_	0.3593	0.0196	0.0540
*AAF*	0.1797	0.0644	0.0886
*PD*	0.1977	0.3779	0.5722
*BM*	0.0687	0.1625	0.0855
*SF*	0.0308	0.1074	0.0254
*AF*	0.0546	0.1196	0.0500
*AD*	0.1092	0.1486	0.1243

Regarding the pile position, *Lx*/*L* = 0.0 is the optimal pile position , as shown in Fig. 21. However, this evaluation result is incorrect, since it can be seen from the simulation results of "Influence of the pile position on the reinforcement effect" section that the deformations of both the slope and anchor are serious when *Lx*/*L* = 0.0; additionally, the anti-slide pile does not fully play its role, indicating that *Lx*/*L* = 0.0 is not the optimal pile position. Moreover, Fig. 21 shows that the reinforcement effect of the pile in the middle and lower part of the slope is better than that in the middle and upper part of the slope, which is consistent with the results presented in "Comparison of the evaluation results" section.

Regarding the pile length and anchor length, the reinforcement effect is poor when the anti-slide pile and anchor are shorter, and there exists an optimal length for both of them. Regarding the anchor angle, the reinforcement effect is poor when the anchor angle is extremely large or extremely small; considering the case of slope considered in this study, 15° is the optimal anchor angle, which is consistent with the results presented in "Comparison of the evaluation results" section.

Regarding the anchor row spacing, the new evaluation results in Fig. 21 show that 6 m is the optimal anchor row spacing; however, this is incorrect, because it can be seen from "Influence of the anchor row spacing on the reinforcement effect" section that when the anchor row spacing is 6 m, the upper part of the slope produces a large deformation and the reinforcement effect is poor.

In summary, there are some obvious errors in the evaluation results when the maximum displacement of slope is considered as the evaluation index. This is because the maximum displacement of the slope under various working conditions does not change significantly, and its *COV*is small, leading to a decrease in both the objective weight and comprehensive weight of the displacement index. Thus, the deformation characteristics of the slope cannot be effectively considered in the evaluation. Therefore, it is not recommended to take the maximum displacement of the slope as the displacement index of the *MOE* model in this study. In contrast, it is more reasonable to use the average displacement of the slope as the displacement index of the *MOE* model, as it can fully incorporate the deformation characteristics of the slope into the evaluation.

### Influence of the normalization method of evaluation indexes of the pile-anchor system on the evaluation effect

**Figure 22:**
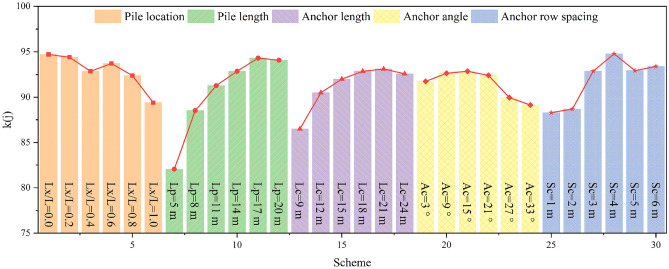
Evaluation results when *BM*, *SF* and *AF* are regarded as intermediate indexes. From left to right, it corresponds to the fuzzy comprehensive evaluation values of the 30 numerical simulation schemes in Table 2. A large fuzzy comprehensive evaluation value indicates a good reinforcement effect.

As depicted in Fig. 1, the reinforcement scheme design of the slope reinforced by the pile-anchor system integrates slope stability, slope dynamic response characteristics and the safety of the pile-anchor system. Undoubtedly, our goal is to ensure the safety of the pile-anchor system to the maximum possible extent under the premise of considering slope stability and slope dynamic response characteristics. To further ensure the safety of the pile-anchor system, the three indexes of *BM*, *SF*, and *AF* are regarded as negative indexes and normalized by Eq. (2) in this section. The new evaluation results are compared with the results of "Comparison of the evaluation results" section, where these three indexes are regarded as intermediate indexes; this is performed to study the impact of the normalization methods of these three indexes on the evaluation results. It should be noted that the values of different types of weights of each index in this section are the same as those in Table 9; additionally, the corresponding evaluation results are illustrated in Fig. 22, from which it can be seen that the new evaluation results are different from the results of "Comparison of the evaluation results" section in some aspects.

Regarding the pile location, when the three indexes of *BM*, *SF*, and *AF* are considered as negative indexes, the reinforcement effect is generally better when piles are placed in the middle and lower parts of the slope, which is consistent with the results of "Comparison of the evaluation results" section. However, it can also be seen from Fig. 22 that *Lx*/*L* = 0.0 is the optimal pile placement; additionally, a poor reinforcement effect was observed at *Lx*/*L* = 0.4; this is inconsistent with the simulation results in "Influence of the pile position on the reinforcement effect" section.

Regarding the pile length, anchor length and anchor angle, when the three indexes of *BM*, *SF*, and *AF* are considered as negative indexes, the corresponding evaluation results are generally consistent with the results in "Comparison of the evaluation results" section. There exists an optimal value for all of these indexes, and the reinforcement effect cannot be further improved after exceeding the optimal value.

Regarding the anchor row spacing, when the three indexes of *BM*, *SF*, and *AF* are considered as negative indexes, the reinforcement effect is the best when the anchor row spacing is 4 m. In other words, 4 m is the optimal anchor row spacing for the case of the slope considered in this study; this is consistent with the results in "Comparison of the evaluation results" section. Additionally, the reinforcement effect is poor when the anchor row spacing is small for both normalization methods, thus indicating that the anchor row spacing should not be extremely small.

To summarize, treating the three indexes of *BM*, *SF*, and *AF* as negative indexes overemphasizes the safety of the pile-anchor system; this may lead to unreasonable evaluation results in some aspects. Considering the strong bending and shearing capacity of the anti-slide pile and the strong tensile capacity of the anchor, we believe that it is more reasonable to regard the three indexes of *BM*, *SF*, and *AF* as intermediate indexes when adopting the *MOE* model; this is conducive to giving full play to the reinforcing capacity of the pile-anchor system, and the corresponding evaluation results closely align with the numerical simulation outcomes.

## Conclusion

This study established a numerical model of bedding rock slope reinforced by a pile-anchor system, analyzed the effects of pile position, pile length, anchor length, anchor angle and anchor row spacing on the seismic reinforcement effect and the safety of the pile-anchor system, proposed a new multi-objective optimization evaluation model of the seismic performance of a slope reinforced by a pile-anchor system, and discussed the impact of the methods of obtaining displacement index and normalizing indexes on the evaluation results. The following conclusions are drawn:

1. In the process of moving the pile position from the foot to the top of slopes, the reinforcement effect first increases and subsequently decreases. If the length of the pile and anchor is extremely small, it may lead to the overall instability of the slope; however, there are optimal values for anchor and pile lengths. An extremely small anchor angle will not allow the pile-anchor system to be fully utilized, while an extremely large anchor angle will increase the danger of the anchor. When the anchor row spacing is extremely small, the reinforcement range is limited; when the anchor row spacing is extremely large, large deformation may occur in the upper part of the slope.
2. The selection of *DIS*, *PD*, *BM*, *SF*, *AF*, *AD*, and *AAF* as optimization evaluation indexes can better evaluate the seismic reinforcement effect of the pile-anchor system. It is found that the method of obtaining the displacement index greatly influences the evaluation results. When *DIS*_*max*_ is used as the displacement index, the deformation characteristics of the slope are easily ignored, which may lead to unreasonable evaluation results. The average displacement of the slope (*DIS*) is an ideal displacement index that fully incorporates the deformation characteristics of the slope into the evaluation and leads to a more reliable evaluation result.
3. The normalization method of the evaluation index greatly influences the evaluation results. If *BM*, *SF*, and *AF* are negative indexes, the safety of the pile-anchor system is overemphasized, leading to unreasonable evaluation results. Thus, *BM*, *SF*, and *AF* should be regarded as intermediate indexes. In this case, the reinforcement effect of the pile-anchor system is fully utilized, and the evaluation result is more reliable.

It should be pointed out the newly-proposed evaluation model has not been verified due to the inaccessibility of the relevant on-site monitoring data of the slope prototype corresponding to the numerical model. Therefore, in the follow-up study we will try to look for some practical engineering cases to verify our evaluation model and then continuously to improve it. Meanwhile, we hope that the concept and framework of our evaluation model can inspire readers so that they can apply our method to their own practical engineering cases and provide feedback on its effectiveness.

## Data Availability

All data generated or analysed during this study are included in this published article.

## List of symbols

*MOE*: Multi-objective optimization evaluation
*TD*: Target design
*P-AS*: Pile-anchor safety
*DR*: Dynamic response
*SS*: Slope stability
*DIS*: The mean displacement of the slope
*PD*: Pile displacement
*BM*: Pile bending moment
*SF*: Pile shear force
*AF*: Anchor axial force
*AD*: Anchor displacement
*AAF*: Acceleration amplification factor
*COV*: Coefficient of variation
*Δl*: Meshing element size
*λ*: The wavelength corresponding to the highest-component frequency of the input wave
*Lx*: The distance from the slope foot to the anti-slide pile
*Lp*: The length of the pile
*Lc*: The length of the anchor
*Ac*: The angle of the anchor
*Sc*: The row spacing of the anchor
*λ*_*max*_: The maximum eigenvalue of judgment matrix
*RI*: Random index
*CI*: Consistency index
*CR*: Consistency ratio
*DIS*_*max*_: The maximum displacement of the slope
